# Lysine-Rich Extracellular Rings Formed by hβ2 Subunits Confer the Outward Rectification of BK Channels

**DOI:** 10.1371/journal.pone.0002114

**Published:** 2008-05-07

**Authors:** Maorong Chen, Geliang Gan, Ying Wu, Lu Wang, Yingliang Wu, Jiuping Ding

**Affiliations:** 1 Key Laboratory of Molecular Biophysics, Huazhong University of Science and Technology, Ministry of Education, College of Life Science and Technology, Wuhan, Hubei, China; 2 State Key Laboratory of Virology, College of Life Sciences, Wuhan University, Wuhan, Hubei, China; University of Texas Austin, United States of America

## Abstract

The auxiliary β subunits of large-conductance Ca^2+^-activated K^+^ (BK) channels greatly contribute to the diversity of BK (mSlo1 α) channels, which is fundamental to the adequate function in many tissues. Here we describe a functional element of the extracellular segment of hβ2 auxiliary subunits that acts as the positively charged rings to modify the BK channel conductance. Four consecutive lysines of the hβ2 extracellular loop, which reside sufficiently close to the extracellular entryway of the pore, constitute three positively charged rings. These rings can decrease the extracellular K^+^ concentration and prevent the Charybdotoxin (ChTX) from approaching the extracellular entrance of channels through electrostatic mechanism, leading to the reduction of K^+^ inflow or the outward rectification of BK channels. Our results demonstrate that the lysine rings formed by the hβ2 auxiliary subunits influences the inward current of BK channels, providing a mechanism by which current can be rapidly diminished during cellular repolarization. Furthermore, this study will be helpful to understand the functional diversity of BK channels contributed by different auxiliary β subunits.

## Introduction

Large-conductance Ca^2+^-activated K^+^ (MaxiK or BK) channels consisting of a tetramer of mSlo1 α subunits are almost ubiquitously expressed among mammalian tissues. They play a critical role in modulating contractile tone of smooth muscle and neuronal process[1]–[4]. However, this tetramer in many tissues often associates with auxiliary β subunits, which produces the diversity of BK channels and suits different roles in a large variety of physiological process. The β subunits increase the apparent Ca^2+^ and voltage sensitivities of mSlo1 α channels, modify the channel kinetics and alter their pharmacological properties [5]–[13]. These regulatory subunits share a putative membrane topology, which contains two transmembrane segments connected by a large extracellular loop (112–123 residues), an intracellular N-terminus and a C-terminus. The β2 subunit of a four-member β family has been found in the rat chromaffin cells, pancreatic β cells and DRG neurons [12], [14], [15]. The hydrophobic N-terminus of β2 subunits induces rapid inactivation of BK channels [13], and its extracellular segment prevents the scorpion toxin Charybdotoxin (ChTX) from approaching the channel pore [12].

Additionally, the β2 like the β3b subunits possesses a nonlinearity on instantaneous current-voltage (I-V) curve of the resulting BK currents, termed rectification [16]. In the redox-experiments, Zeng et al postulated that elements of β3b subunits resided sufficiently close to the pore, essentially forming a new type of gate to ion permeation [16]. It provides us an interesting and novel view for understanding the modulatory mechanism of β subunits. However, it is unclear what and where the functional element is, and how it can confer the outward rectification of BK current. Here, we seek to further elucidate the outward rectification mechanism defined by the hβ2 extracellualr segment.

## Results

### The hβ2 auxiliary subunit alters the property of rectification of BK channels

**Figure 1:**
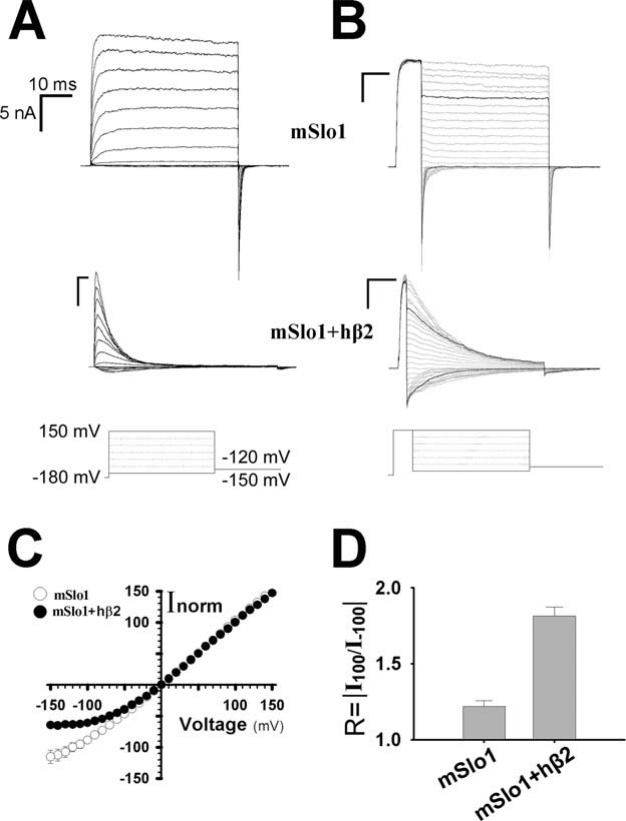
The instantaneous current-voltage properties of mSlo1 and mSlo1+hβ2 channels. A. Traces show the currents recorded from inside-out patches from HEK293 cells transfected with mSlo1 alone (top panel) or mSlo1+hβ2 (middle panel). Currents were elicited at potentials from −150 through 150 mV in an increment of 20 mV, after a prepulse to −180 mV to remove inactivation, in the presence of 160 mM K_o_^+^/160 mM K_i_^+^ with 10 µM Ca^2+^. The voltage protocol is plotted at the bottom panel. Scale bars represent 10 ms and 5 nA, respectively. B. Traces were obtained from the same patch as shown at the left panel in A. Instantaneous tail currents of mSlo1 and mSlo1+hβ2 were activated by steps to voltages ranging from −150 to 150 mV with an increment of 10 mV after a 5 or 10 ms prepulse of 150 mV, in the presence of 10 µM Ca^2+^. The voltage protocol is plotted at the bottom. The dark lines represent the currents activated at +100 mV and −100 mV, respectively. Scale bars represent 10 ms and 5 nA, respectively. C. The instantaneous tail currents of mSlo1 (empty circle) and mSlo1+hβ2 (solid circle), after normalized to the tail current at +100 mV, were plotted as function of voltages. D. The rectification ratios R = |I_100_/I_−100_| were plotted for mSlo1 and mSlo1+hβ2. They are 1.22±0.04 (n = 8) and 1.81±0.06 (n = 17) for mSlo1 and mSlo1+hβ2, respectively.

BK channels encoded with mSlo1 α subunits show a rapid inactivation behavior after associated with its auxiliary hβ2 subunits [11], [12]. In Fig. 1A, the representatives of the mSlo1 and mSlo1+hβ2 currents were recorded from an inside-out patch under symmetrical 160 mM K^+^ solution with the intracellular application of 10 µM Ca^2+^. The BK channels composed of mSlo1 and hβ2 can have up to four hβ2 subunits, which can be achieved by adding the excessive amount of hβ2 plasmid during transfection. The BK channel associated with four hβ2 subunits has a half-maximal activation voltage V_50_≈−15 mV with inactivation time constant τ_i_≈20 ms at 100 mV, in the presence of 10 µM Ca^2+^ [17], [18]. In this study, all the currents were derived from the mSlo1+hβ2 channels with a full complement of hβ2 subunits, which were judged by their values of V_50_ or τ_i_ (See the Materials and Methods). Using symmetrical 160 mM K^+^ solutions, the tail currents of the mSlo1 and the mSlo1+hβ2 were activated by a voltage step to −180 mV, followed by steps to potentials from –150 to 150 mV, in the presence of 10 µM Ca^2+^ (Fig. 1B). The normalized instantaneous tail currents were plotted as a function of repolarization potential for the mSlo1 and the mSlo1+hβ2 (Fig. 1C). In contrast to the mSlo1 α channel alone, the mSlo1+hβ2 channel shows nonlinearity on its instantaneous current-voltage (I-V) curve, which is similar to that of mSlo1+β3b [16], [19]. Normalized instantaneous I-V curves were used to determine the property of rectification of mSlo1+hβ2 channels. To quantitatively describe the nonlinearity behavior, we introduced a term i.e. rectification ratio R = |I_100_/I_−100_|. From the traces (±100 mV) in thick black lines shown in Fig. 1B, we found that the rectification ratios (R) were 1.22±0.04 for mSlo1 and 1.81±0.06 for mSlo1+hβ2 (Fig. 1D).

Those results are consistent with the previous report, i.e. neither the positively charged molecular (such as Mg^2+^) nor the cytosolic NH_2_ or COOH terminus of hβ2 is related to the nonlinearities in this case [16]. Thus, the instantaneous outward rectification of mSlo1+hβ2 should be the intrinsic characteristic of channels derived from the extracellular loop of hβ2. It is known that the β1 and β4 subunits do not alter the property of rectification of mSlo1 channels as the β2 and β3b subunits do [16]. Therefore, if the external loops of β subunits are exchanged between two different members of the β family, the phenotypes regarding the rectification will correspond to their respective loops. As replacements of smaller segments of the loops by chimeras failed to identify a discrete locus responsible for rectification [16], we were facing a provocative question: which portion of the hβ2 extracellular loop is the functional domain.

### Lysine-rich domain of the extracellular loop of hβ2 subunits is fundamental to rectification

Intuitively, we inferred that it should be near the entrance of the extracellular pore. However, it is not easy to locate the functional domain of the hβ2 extracellular loop since the loop is huge. Fortunately, our previous work regarding the surface trafficking of hβ2 provide an important clue for determining the functional region in the hβ2 external loop [20]. The evidence arose out of a c-myc epitope, i.e. ***EQKLISEEDL***, tagged at the position 137 of hβ2, which surprisingly eliminated the rectification of mSlo1+hβ2 channels completely. It suggested that the extracellular functional locus might be positioned at the central region of the hβ2 external loop from the position 137 through the position 147. In contrary to the glutamate-rich (acidic) sequence of the c-myc epitope, the 137–147 segment of hβ2 has a lysine-rich (basic) sequence, i.e. ***KINQKCSYIPK***. It seems reasonable that the positively charged rings formed by those lysines influence the single-channel conductance of channels as the negatively charged rings located around the mSlo1 pore do [21], [22]. We thus preferred to suppose that positive charges might play a crucial role in rectification.

**Figure 2:**
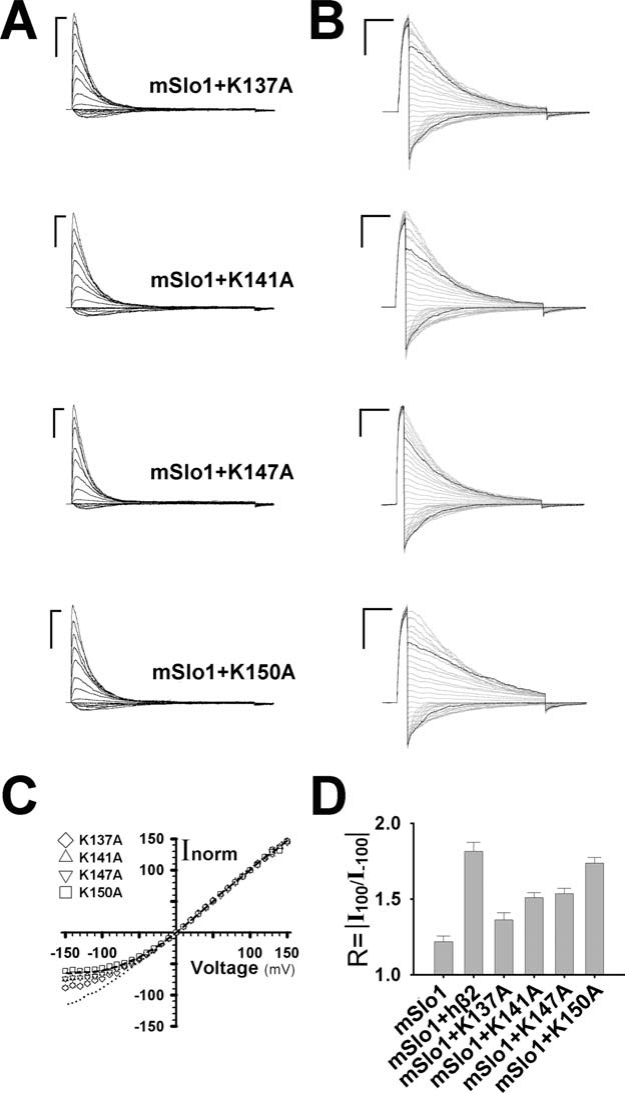
Basic amino acids of hβ2 extracellular domain contribute to the outward rectification of BK channels. A. The representative traces are for mSlo1+K137A, mSlo1+K141A, mSlo1+K147A and mSlo1+K150A as indicated. The voltage protocol is the same as shown in Fig. 1A. Scale bars represent 10 ms and 5 nA, respectively. B. The instantaneous tail currents from the top to the bottom are for mSlo1+K137A, mSlo1+K141A, mSlo1+K147A and mSlo1+K150A, respectively. The voltage protocol is the same as described in Fig. 1B. Scale bars represent 10 ms and 5 nA, respectively. C. The instantaneous I-V curves normalized to the tail currents at +100 mV are for mSlo1+K137A, mSlo1+K141A, mSlo1+K147A and mSlo1+K150A as indicated. The long dash and the dotted line are for mSlo1+hβ2 and mSlo1, respectively. D. The rectification ratios R = |I_100_/I_−100_| were plotted for mSlo1, mSlo1+hβ2, mSlo1+K137A, mSlo1+K141A, mSlo1+K147A and mSlo1+K150A as indicated. They are 1.22±0.04 (n = 8) and 1.81±0.06 (n = 17), 1.36±0.05 (n = 11), 1.51±0.03 (n = 15), 1.53±0.04 (n = 14) and 1.74±0.04 (n = 12) for mSlo1, mSlo1+hβ2, mSlo1+K137A, mSlo1+K141A, mSlo1+K147A and mSlo1+K150A, respectively.

After carefully checking, we found there were actually four lysines surrounding the central region of extracellular loop of hβ2. To examine whether they can induce the rectification of mSlo1+hβ2 channels, four lysines were neutralized to alanine individually. In Fig. 2A, all of mSlo1+mutation channels had the stoichiometry of α:β = 1:1 because they showed a V_50_≈−15 mV or τ_i_≈20 ms (See the Materials and Methods). Instantaneous currents showed that all of them can partially reduce the outward rectification ratio (Fig. 2B). The neutralization of lysine (K) reduces the value of R from 1.8 to 1.35 for K137A, 1.51 for K141A, 1.53 for K147A and 1.74 for K150A (Fig. 2C–D). The variation of R reflects that distances between the pore center and each lysine are unequal. Obviously, the site 137 with the minimal R is the closest site to the pore center of channel, both the sites 141 and 147 with the medium R are equally the farther from the center, and the site 150 with the maximal R is the farthest to the center pore probably just on the verge of the functional region.

### Rectification behavior has additivity

**Figure 3:**
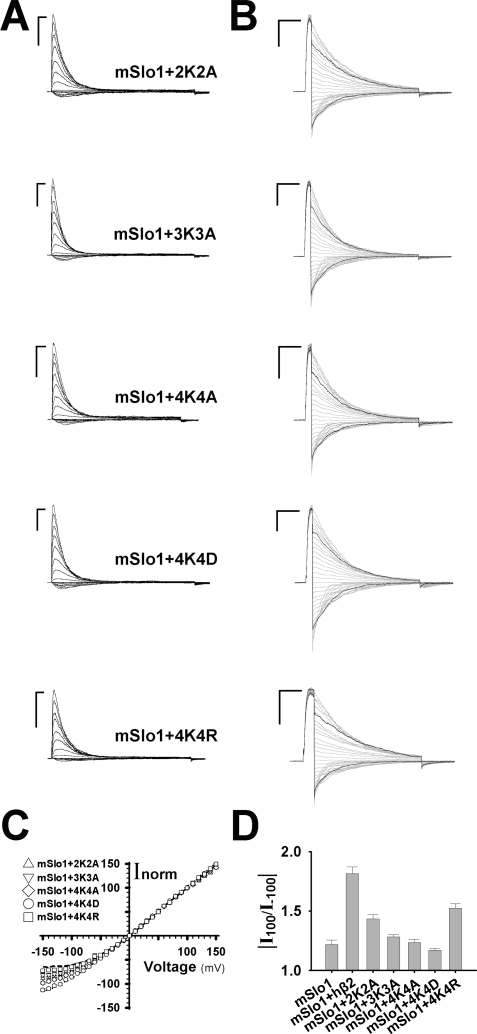
The multi-lysine mutations of hβ2 alleviate the outward rectification of BK channels. A. The representative traces are for mSlo1+2K2A, mSlo1+3K3A, mSlo1+4K4A and mSlo1+4K4D, respectively. Here, the 2K2A, 3K3A, 4K4A and 4K4D are short for K137AK141A, K137AK141AK147A, K137AK141AK147AK150A and K137DK141DK147DK150D, respectively. The voltage protocol is the same as shown in Fig. 1A. Scale bars represent 10 ms and 5 nA, respectively. B. The instantaneous tail currents from the top to the bottom are for mSlo1+2K2A, mSlo1+3K3A, mSlo1+4K4A, mSlo1+4K4D and mSlo1+4K4R, respectively. The voltage protocol is the same as described in Fig. 1B. Scale bars represent 10 ms and 5 nA, respectively. C. The instantaneous I-V curves normalized to the tail currents at +100 mV are for mSlo1+2K2A, mSlo1+3K3A, mSlo1+4K4A, mSlo1+4K4D and mSlo1+4K4R as indicated. The long dash and the dotted line are for mSlo1+hβ2 and mSlo1, respectively. D. The rectification ratios R = |I_100_/I_−100_| were plotted for mSlo1, mSlo1+hβ2, mSlo1+2K2A, mSlo1+3K3A, mSlo1+4K4A, mSlo1+4K4D and mSlo1+4K4R as indicated. They are 1.22±0.04 (n = 8) and 1.81±0.06 (n = 17), 1.43±0.04 (n = 15), 1.28±0.02 (n = 12), 1.24±0.03 (n = 13), 1.17±0.01 (n = 13) and 1.55±0.03 (n = 12) for mSlo1, mSlo1+hβ2, mSlo1+2K2A, mSlo1+3K3A, mSlo1+4K4A, mSlo1+4K4D and mSlo1+4K4R, respectively.

It is well known that electrostatic field has additivity. To test whether the rectification effect (or R) has additivity, we changed the number of net charges in the lysine-rich region. In other words, decrease in the number of positive charges of the lysine-rich region should dent the nonlinearity of instantaneous I-V curves or reduce the value of R. To validate this hypothesis, the double (K137AK141A, termed 2K2A), triple (K137AK141AK147A, termed 3K3A) and quadruple (K137AK141AK147AK150A, K137DK141DK147DK150D and K137RK141RK147RK150R, termed 4K4A, 4K4D and 4K4R respectively) mutations were constructed, and their rectification ratios were then measured. The change in the number of elementary charges (Δq_e_), for instance, will be −2 for one subunit of the mutant 2K2A, and −8 for the whole channel. In this study, Δq_e_ represents the change in the number of net charge of the whole channel, compared with the number of charges of hβ2. As mentioned previously, Fig. 3A indicated that the stoichiometry of mSlo1+hβ2-mutant channels was always kept at α:β = 1∶1, which were judged by their values of V_50_ or τ_i_ (See the Materials and Methods). With the Δq_e_ decreased from 0 to –32, the instantaneous I-V curve was gradually off the I-V curve of α+hβ2 until it reached the I-V curve of mSlo1 alone (Fig. 3B–C). Correspondingly, the rectification ratio (R_mut_) of mutant gradually descended to the ratio (R_mSlo1_) of mSlo1 alone or even went beyond it, while the Δq_e_ decreased from 0 (hβ2) to –32 (4K4D), indicating that the rectification behavior has additivity (Fig. 3D). To further confirm the electrostatic effect, the mutant 4K4R was then constructed. Even though the mutant 4K4R has V_50_≈3.8 mV, indicating there is a structural change probably due to the larger volume of Arginine, it still has an inactivation time constant τ_i_≈16 ms (See the Materials and Methods). The rectification ratio of 4K4R is about R = 1.6, suggesting that its basic feature of rectification has not been changed by mutation (Fig. 3A–D). In this study, we found that all the hβ2 mutations except the mutation 4K4R largely shared most of kinetic characteristics in activation, inactivation and recovery with the wild-type hβ2 subunits, suggesting that the net charge rather than the structural change confers the nonlinearity on instantaneous I-V curves.

### The lysine-rich extracellular rings of hβ2 reduce the single-channel conductance

**Figure 4:**
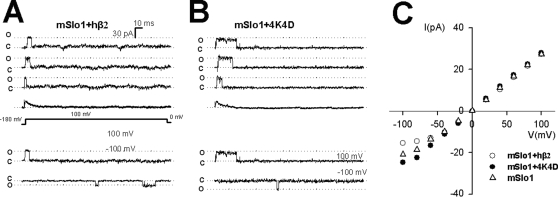
Single-channel conductance of mSlo1+hβ2 and mSlo1+4K4D. A–B, Three consecutive traces at the upper panel show the single-channel openings from inside-out patches from *Xenopus oocytes* injected with the cRNA encoding mSlo1+hβ2 or mSlo1+4K4D subunits as indicated. Channels were activated by a voltage step to +100 mV after a prepulse to −180 mV to remove inactivation, in the presence of 10 µM Ca^2+^. Average traces of each channel derived from 50 sweeps are shown below the traces. The inactivation time constants of mSlo1+hβ2 and mSlo1+4K4D are 10.9 ms and 10.1 ms at +100 mV, respectively. Single-channel openings at a voltage of +100 mV and −100 mV are shown in the lower panel. D, Single-channel currents were plotted as function of membrane potentials for mSlo1+hβ2 (empty circle), mSlo1+4K4D (solid circle) and mSlo1 (empty triangle). The rectification ratios (R) of mSlo1+hβ2 and mSlo1+4K4D are 1.81±0 .05 (n = 4) and 1.13±0.05 (n = 4), respectively.

To determine whether net charge of the rings of lysines can affect the single-channel conductance, we examined the single-channel currents of mSlo1+hβ2 and mSlo1+4K4D. Representatives of single-channel currents from oocytes expressing the above channels are shown in Fig. 4A–B. All single-channel experiments are performed by over-expressing the hβ2 and 4K4D to ensure the 1∶1 stoichiometry, which can be estimated by the inactivation time constants [12], [17], [18]. Fitting to the assembled single-channel currents, we found that both currents had an equal inactivation time constant of τ_i_≈10 ms at 100 mV, indicating that there were four β subunits per channel in those experiments. The mSlo1+hβ2 showed strong outward rectification with R = 1.8 in the single-channel currents, whereas the mSlo1+4K4D showed much less rectification with R = 1.1 (Fig. 4A–C). In Fig. 4C, the single-channel conductance of mSlo1+4K4D (Δq_e_ = −32), mSlo1 (Δq_e_ = −16) alone and mSlo1+hβ2 (Δq_e_ = 0) is the largest, the second largest and the smallest at negative potentials, respectively, which is closely consistent to the results of macroscopic recordings.

### Lysine-rich segment of hβ2 reduces the Charybdotoxin (ChTX) sensitivity of BK channels

Using the c-myc epitope tagged the extracellular loop of hβ2, we found that the most accessible site by antibody was located at the middle of the extracellular loop, suggesting that that region might be very important in regulation of the toxin sensitivity and the rectification of BK-type channels [20]. To test this hypothesis, all the basic residues (Lysine) in the region were substituted for the acidic residues (Aspartate). In Fig, 5, the wild type hβ2 shows much less sensitivity (τ_on_ = 138.3±16.9 s, n = 4) than that of the mutant 4K4D (τ_on_ = 20.00±1.3 s, n = 4), which is consistent with the above hypothesis. It demonstrates that four lysines play a critical role in both the sensitivity and rectification of channels, suggesting that the lysine-rich segment is lying at the outer entrance of BK channels. Although the extracellular structure of each β (β1–β4) can be very different, there is only one thing in common: Electrostatic effect.

**Figure 5:**
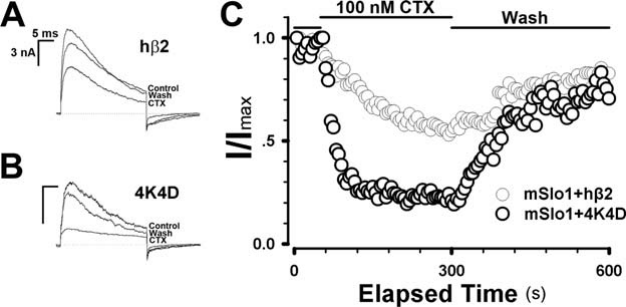
The extracellualr segment of hβ2 affects the sensitivity of ChTX to mSlo1+hβ2 channels. A–B, Traces show the representative currents obtained from outside-out patches from HEK293 cells transfected with cDNA encoding mSlo1+hβ2 and mSlo1+4K4D subunits, respectively. Currents were elicited by a 30-ms voltage step from –180 mV to 120 mV, in the presence of 10 µM Ca^2+^. The current traces are for the control, 100 nM ChTX and recovery as indicated. C. The time courses of blockade of mSlo1+hβ2 and mSlo1+4K4D by 100 nM ChTX. Each patch was perfused with 100 nM ChTX as indicated by the horizontal bars. The on-time constants of blockade by 100 nM ChTX are: τ_on_ = 138.3±16.9 s (n = 4) for mSlo1+hβ2 (gray circle) and τ_on_ = 20.0±1.3 s (n = 4) for mSlo1+4K4D (black circle).

### The outward rectification induced by the electrostatic field from the hβ2 extracellular loop

**Figure 6:**
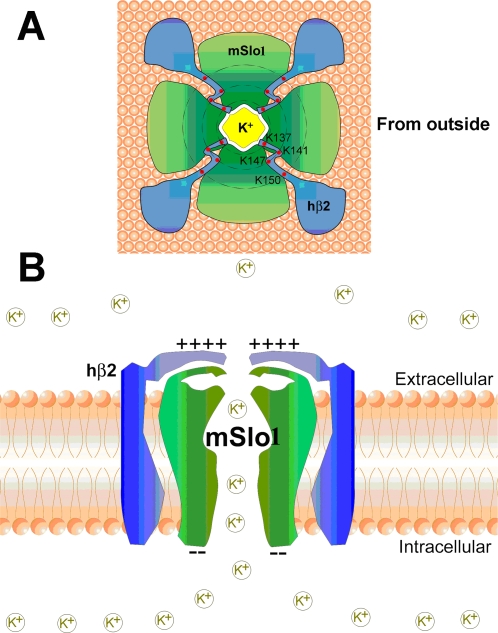
A cartoon for arrangement of the positively charged rings of the hβ2 extracellular domain. A, The conformation of mSlo1+hβ2 viewed from the top. B, The conformation of mSlo1+hβ2 viewed from the lateral side.

Based on the above results, we conclude that the rings of positive charged lysines formed by the hβ2 extracellualr segment confer the outward rectification of mSlo1+hβ2 channels. The positive charges can attenuate the local K^+^ concentration at the entrance of the extracellular vestibule through an electrostatic mechanism [21], [22]. To explain it more clearly, a cartoon diagram shows three rings of positive charges surrounding the entrance of BK channels (Fig. 6A–B). The inner ring is composed of four K137 residues, and the intermediate ring is composed of eight lysines including four K141 and four K147, whereas the outer ring composed of four K150 residues is just located at the edge of the pore (Fig. 6B). Those rings would decrease the possibility of K^+^ being in the extracellular entrance, and thereby, decrease the possibility of K^+^ passing through the BK channel filter to reduce single-channel conductance of BK channels.

## Discussion

The β auxiliary subunits modulate the Ca^2+^ sensitivity, pharmacological property and gating kinetics of BK channels, greatly contributing to BK channel diversity, which is fundamental to adequate function in many tissues. Preliminary studies reveal that the extracellular loop of the β auxiliary subunits determines the toxin binding and closely faces the pore [8]. In this study, we reveal that the outward rectification of BK channel is caused by the positively charged rings of hβ2 external loop located at the outer entrance of the channel pore. Under the condition of the outward rectification, the current traces at the negative voltages, especially in the higher Ca^2+^ probably larger than 10 µM, are apparently much smaller than the values used to be, which can somehow change the G-V curve. The nonlinear instantaneous I-V curve can be used in modifying the current traces to give much better results during a modeling work. Additionally, the reduction of inward currents due to the outward rectification of BK channels can facilitate the cellular repolarization [16].

There are several mechanisms possibly leading to the rectification: the redox-sensitive extracellular gate [16]; the disruption of cooperativity and low open probability [23], [24]; the electrostatic effect [25]. Imoto et al (1998) reported that rings of negatively charged residues lining the entryway into the pore enhanced the conductance of the nicotinic acetylcholine receptor [25]. And recent work showed that the negatively charged residues in both the inner and outer vestibule of the BK (mSlo1) channels could markedly affect the channel conductance [21], [26]. In this study, we demonstrate that the electrostatic field, which is produced by three positively charged rings of hβ2 near the pore, confers the outward rectification of the mSlo1+hβ2 channels.

Our previous work has revealed that the region near the position 137 is the only region of the external loop exposing to the outside [20], implying that this extracellular region is open widely for the bigger molecule to access. As electrostatic field has additivity, total electrostatic field at the above region is the sum of fields contributed by each lysine of the hβ2 loop. However, it is difficult to calculate the exact field at that region due to lack of the information regarding the location of each lysine. In other words, we cannot get the exact K^+^ concentration at that region, although the information about the local K^+^ concentration is so important for understanding the rectification mechanism. Thus, the crystal structure of the hβ2 loop is required for the future study.

The mSlo1+hβ2 channel exhibits toxin-resistance, which can arise from two mechanisms: limited space and Coulomb repulsion. A “helmet” formed by both the turret of mSlo1 and the external loop of hβ2 can prevents toxin from approaching the channel pore [12], and the lysine-rich extracellular segment of hβ2 repulses the lysine-rich ChTX, which results in the low binding affinity of ChTX to BK channels.

## Materials and Methods

### Molecular Biology

Full length cDNA for mSlo1 (Accssion Number NP_034740) and hβ2 (Accssion Number NP_852006) were subcloned into pcDNA3.1Zeo (+) (Invitrogen) and pIRES2-EGFP (BD Biosciences Clontech), respectively. Mutations were obtained by the QuickChange Site-Directed Mutagenesis Kit (Stratagene). In order to get the cRNA of hβ2 and the mutants, cDNA of hβ2 and mutants were subcloned into pXMX (a vector kindly provided by Lingle C.J.). All of the constructs were checked by sequencing. To prepare ^M7^GppGp-capped cRNA, the plasmids were linearized with Enzyme MluI and then in vitro transcribed by SP6 RNA polymerase (Roche). mSlo1 and hβ2 cRNA (diluted to ∼50 ng/ul) were coinjected into Xenopus laveis oocytes (Stage IV) for electrophysiological studies. The ratio of α- to β- subunit cRNA for injection was 1∶2, ensuring the large molar excess of β-subunit RNA.

### Cell culture and Transfection

HEK293 cells were cultured in modified Eagle's medium (DMEM, Gibco) supplemented with 10% fetal bovine serum (FBS, Gibco) at 37°C incubator with 5% CO2. The day before transfection, cells were transfered to a 24-well plate and transiently transfected using lipofectamine2000 (Invitrogen) according to manufacturer̀s protocol. And for co-transfection, the ratio of α- to β- subunit plasmid is 1∶2. Recordings were carried out in 1–2 days after transfection.

### Solutions

Cells were maintained in ND-96 solution (PH 7.5) containing the following (In mM): 96 NaCl, 2 KCl, 1.8 CaCl_2_, 1 MgCl_2_, 2.5 sodium pyruvate and 10 HEPES without penicillin. For inside-out recording, the pipette solution contained the following (in mM): 160 MeSO_3_K, 2 MgCl_2_, 10 HEPES (PH 7.0) titrated with MeSO_3_H. Puff solution contained the following (in mM): 160 MeSO_3_K, 10 HEPES, 5 N-hydroxyethylenediaminetriacetic acid (HEDTA) with added Ca^2+^ to make 10 µM free Ca^2+^, as defined by the EGTAETC program (McCleskey, Vollum Institute, Portland, OR), with the pH adjusted to 7.0. Zero Ca^2+^ solution for puff contained the following (in mM): 160 MeSO_3_K, 10 HEPES, 5 EGTA with the PH 7.0, titrated by MeSO_3_H. All the chemicals were attained from Sigma.

### Electrophysiology

Patch pipettes pulled from borosilicate glass capillaries with resistance of 2–4 megohms when filled with pipette solution. Macroscopic currents were obtained an inside-out patch by excision from the transfected HEK293 cells. Experiments were performed using a PC2C patch-clamp amplifier and corresponding software (InBio, China). Currents were typically digitized at 100 kHz. Macroscopic records were filtered at 5 kHz. During recording, the corresponding solution was puffed onto cells via a puffer pipette containing seven solution channels. Single-channel currents were recorded in inside-out patches from BK channels expressed in oocytes. For single-channel recording, data were acquired with an Axopatch 200A amplifier (Axon Instruments, Foster City, CA), sampled every 3 µs by using a Digidata 1200A (Axon Instruments) and PCLAMP7, and further analyzed with custom software. The single-channel recordings were sampled at 20 KHz and filtered to 10 KHz. Single-channel current amplitudes were analyzed by the QuB software package (State University of New York at Buffalo). All experiments were performed at 21–23°C.

### Data analysis

Recording data were analyzed with IGOR (Wavemetrics, Lake Oswego, OR), Clampfit (Axon Instruments, Inc.) and Sigmaplot software (SPSS, Inc.). Unless stated otherwise, the data are presented as mean±S.D.

For the instantaneous I-V curves of mSlo1+hβ2, the values of the tail currents were measured at 0.25 ms from the starting time point of deactivation. The rectification ratio of mSlo1+hβ2 channels defined as R = I(100 mV)/I(−100 mV) is almost constant for hβ2 at the 0.1–0.5 ms, which is similar to the results reported by Zeng et al. [16]. The stoichiometry of α:β was determined by measuring the half-maximal activation voltage (V_50_) or inactivation time constant (τ_i_) at 100 mV, in the presence of 10 µM Ca^2+^. The averages of V_50_ and τ_i_ (n>10) are: −13.5±2.6 mV and 17.0±0.5 ms for hβ2; −11.2±9.5 mV and 15.5±0.4 ms for K137A; −12.6±6.2 mV and 16.6±0.8 ms for K141A; −12.5±2.0 mV and 13.2±0.5 ms for K147A; −10.5±5.1 mV and 15.9±0.9 ms for K150A; −10.9±3.0 mV and 14.6±0.6 ms for K137A141A; −10.4±2.7 mV and 13.7±0.8 ms for K137A141AK147A; −11.5±2.7 mV and 12.9±0.7 ms for K137A141AK147AK150A; −17.5±1.7 mV and 13.4±0.5 ms for K137DK141DK147DK150D and 3.8±1.9 mV and 16.1±1.3 ms for K137RK141RK147RK150R, respectively.
